# Antimicrobial-resistant gonorrhoea: the national public health response, England, 2013 to 2020

**DOI:** 10.2807/1560-7917.ES.2022.27.40.2200057

**Published:** 2022-10-06

**Authors:** Rachel Merrick, Michelle Cole, Rachel Pitt, Qudsia Enayat, Zdravko Ivanov, Michaela Day, Suzy Sun, Katy Sinka, Neil Woodford, Hamish Mohammed, Helen Fifer

**Affiliations:** 1 United Kingdom Health Security Agency, London, United Kingdom

**Keywords:** antimicrobial resistance, gonorrhoea, surveillance, treatment

## Abstract

*Neisseria gonorrhoeae* has developed resistance to all antimicrobials used to treat gonorrhoea, and the emergence of ceftriaxone-resistant strains threatens the last-line option for empirical treatment. The 2013 Gonococcal Resistance to Antimicrobials Surveillance Programme (GRASP) Action Plan recommended measures to delay the spread of antimicrobial resistance (AMR) in *N. gonorrhoeae* in England. We reviewed trends in gonococcal AMR since then and the experience of implementing the Action Plan’s recommendations to respond to incidents of resistant *N. gonorrhoeae*. Between 2013 and 2019, diagnoses of gonorrhoea in England rose by 128% to 70,922, the largest annual number ever reported. Over this period, *N. gonorrhoeae *isolates have become less susceptible to azithromycin (minimum inhibitory concentration > 0.5 mg/L), increasing from 4.7% in 2016 to 8.7% in 2020; this led to a change in first-line treatment for gonorrhoea in the United Kingdom (UK) from dual therapy (ceftriaxone/azithromycin) to ceftriaxone monotherapy in 2019. We also detected the first global treatment failure for pharyngeal gonorrhoea with a dual-therapy regimen (ceftriaxone/azithromycin), followed by an additional six ceftriaxone-resistant strains. Continued engagement of sexual health clinicians and laboratories with the UK Health Security Agency (UKHSA) is essential for the timely detection of *N. gonorrhoeae* strains with ceftriaxone resistance and to rapidly contain transmission of these strains within England.

## Background

*Neisseria gonorrhoeae*, the pathogen causing gonorrhoea, has developed resistance to all classes of antibiotics recommended for treatment, including to third-generation cephalosporins, the last-line options for empirical monotherapy. Untreated gonorrhoea in women can lead to pelvic inflammatory disease, infertility and ectopic pregnancy. In 2012, the World Health Organisation (WHO) and the European Centre for Disease Prevention and Control (ECDC) both published response plans to control the spread and impact of antimicrobial resistance (AMR) in *N. gonorrhoeae* [1,2]. To support these response plans in England, the Health Protection Agency (HPA) (which became Public Health England (PHE) in 2013 and then the United Kingdom Health Security Agency (UKHSA) in 2021) published in 2013 the Gonococcal Resistance to Antimicrobials Surveillance Programme (GRASP) Action Plan to advise on the national response to gonococcal AMR [3].

The Action Plan’s objectives were to: provide robust and timely surveillance data on gonococcal AMR in England and Wales; advise on appropriate changes to the national guidelines for the management of gonorrhoea; give technical advice to clinical microbiologists on appropriate methods for detection of resistant gonococcal isolates in the laboratory; provide support to allow rapid detection of treatment failures; communicate to relevant healthcare professionals and populations with higher rates of gonorrhoea diagnoses to raise awareness of the threat of untreatable gonorrhoea; and promote prevention messages to enhance public health control of gonorrhoea.

Between 2013 and 2019, reported diagnoses of gonorrhoea rose by 128% in England, from 31,114 to 70,922; the largest number ever reported [4,5]. The emergence of multi-drug resistant *N. gonorrhoeae* strains in recent years is of worldwide concern [6]. Indeed, the *N. gonorrhoeae* FC428 clone, which is associated with ceftriaxone resistance and reduced susceptibility to azithromycin, has been detected in numerous countries globally, usually with epidemiological links to the Asia-Pacific region [7,8]. While sustained transmission of the FC428 clone has not been reported outside of the Asia-Pacific region, the ease with which this strain spread internationally attests to how rapidly gonorrhoea might become untreatable.

Here we describe gonococcal AMR trends, outbreaks and incidents in England since the Action Plan was published in 2013, and how UKHSA has responded by developing and improving surveillance of AMR in England.

## Gonococcal antimicrobial resistance trends

**Figure 1:**
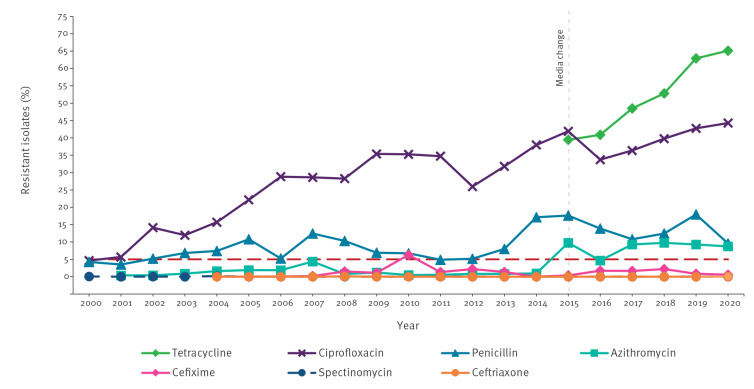
Percentage of *Neisseria gonorrhoeae* isolates in the GRASP sentinel surveillance system that were resistant to selected antimicrobials, England and Wales, 2000–2020

MIC: minimum inhibitory concentration; GRASP: Gonococcal Resistance to Antimicrobials Surveillance Programme.

Since 2000, GRASP has monitored trends in gonococcal AMR and has provided quality-assured data to inform the revision of national clinical gonorrhoea management guidelines. The GRASP sentinel surveillance programme combines antimicrobial susceptibility testing with enhanced epidemiological and behavioural data to detect emerging trends [9]. GRASP has shown evidence of isolates becoming less susceptible to azithromycin (minimum inhibitory concentration (MIC) > 0.5 mg/L), from 4.7% in 2016 to 8.7% in 2020, which helped inform the change in first-line treatment from dual therapy (ceftriaxone plus azithromycin) to ceftriaxone monotherapy in the 2019 UK gonorrhoea treatment guideline [10,11]. Cefixime resistance has remained low for some years, at 0.6% in 2020, but both ciprofloxacin (44.3% in 2020) and tetracycline (65.1% in 2020) resistance have continued to increase despite infrequent use for the treatment of gonorrhoea (Figure 1). Isolates with decreased susceptibility to ceftriaxone (MIC > 0.03 mg/L) increased from 0.3% in 2013 to 7.1% in 2018 but decreased to 1.4% in 2020. It is possible that this decline may be due to the increased dose of ceftriaxone (from 500 mg to 1 g) recommended in the 2019 UK gonorrhoea treatment guideline [11]. In 2019, gentamicin susceptibility testing was added to the GRASP panel; although there is no clinical breakpoint, the low modal MIC (4 mg/L), provided evidence to support the use of gentamicin for treating gonorrhoea. It should be noted that in 2015, the commercial medium used for susceptibility testing in GRASP was changed to a new medium which supported better growth of *N. gonorrhoeae* isolates. Comparison of MICs determined on the old and new media indicated differences for some antimicrobials, most notably tetracycline and azithromycin; MICs of tetracycline decreased, whereas the MICs of azithromycin increased on the new medium. For this reason, MICs for the 2015 collection are not directly comparable to those from previous years, and trends must be interpreted with caution [12].

This figure is reproduced from the GRASP report [10].

Vertical dashed black line: point of change in the diagnostic sensitivity medium used to test antimicrobial susceptibility of sentinel surveillance isolates. MICs for the 2015 to 2020 collections are not directly comparable with those from previous years. Trends from 2000 to 2014 compared with 2015 to 2020 must be interpreted with caution, particularly for azithromycin (MICs higher on the new medium) and tetracycline (MICs lower on the new medium; data for tetracycline are only included from 2015 onwards due to this issue) [12].

Horizontal dashed red line: the 5% threshold (≥ 5% of infections resistant to the first-line therapy) at which the World Health Organization recommends that first-line monotherapy guidelines should be changed.

## Detection and response to antimicrobial resistance incidents

**Figure 2:**
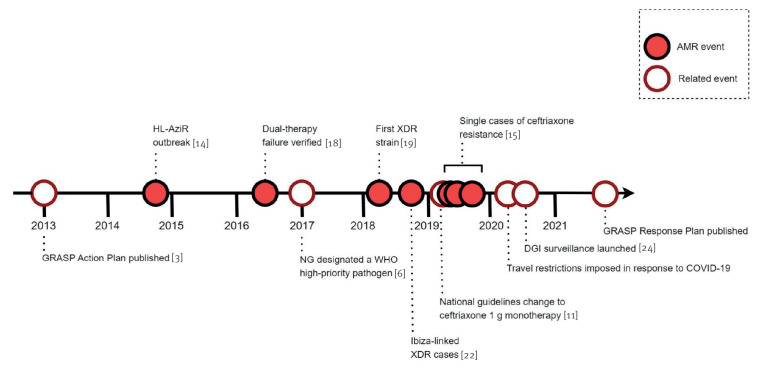
Timeline of incidents with antimicrobial-resistant *Neisseria gonorrhoeae* and related events, England, 2000–2020

AMR: antimicrobial resistance; COVID-19: coronavirus disease; DGI: disseminated gonococcal infection; GRASP: Gonococcal Resistance to Antimicrobials Surveillance Programme; HL-AziR: high-level azithromycin-resistant; NG: Neisseria gonorrhoeae; WHO: World Health Organization; XDR: extensively drug-resistant.

Since the GRASP Action Plan was published in 2013, there have been several incidents with resistant *N. gonorrhoeae* which have required a national public health response (Figure 2).

In response to an outbreak of high-level azithromycin-resistant (HL-AziR, MIC > 256 mg/L) *N. gonorrhoeae* in northern England in 2015, PHE (now UKHSA) issued a national resistance alert [13], advising that primary diagnostic laboratories should test all gonococcal isolates for azithromycin resistance, and gave technical advice regarding methods for determining antimicrobial susceptibility. The national reference laboratory provided a free-of-charge confirmatory testing service for isolates with suspected HL-AziR. Whole-genome sequencing (WGS) revealed that most of the HL-AziR isolates belonged to a single clone, ST 9768 [14]. PHE alerted sexual health clinicians through the British Association for Sexual Health and HIV (BASHH), stating that particularly rigorous follow-up efforts for partner notification and test-of-cure were required for individuals infected with the HL-AziR strain. Despite intensive efforts to notify and test partners, HL-AziR strains spread nationally and into different sexual networks [14], with a total of 245 HL-AziR cases confirmed by the national reference laboratory in 2019 [15]. At the time of the outbreak, only 35% of sexual partners reported by heterosexual cases and no partners reported by gay, bisexual and other men who have sex with men (GBMSM) were confirmed to have been successfully contacted and tested, despite considerable efforts made by clinical staff [16]. While partner notification proved challenging, the proportion of primary diagnostic laboratories referring isolates with suspected azithromycin resistance to the national reference laboratory increased by 16% from 2015 (96/282) to 2017 (219/437). The outbreak received substantial national and local media attention, raising the profile of HL-AziR gonorrhoea and leading to an increase in gonorrhoea-related Internet searches which were spatio-temporally associated with increases in sexual health clinic attendances and gonorrhoea diagnoses [17].

In 2016, the first global treatment failure for pharyngeal gonorrhoea with a dual-therapy regimen (ceftriaxone 500 mg plus azithromycin 1 g) due to a ceftriaxone-resistant strain was detected in England [18]. The case was a heterosexual male who had recently returned from Japan where his female partner had been treated for gonorrhoea. The national reference laboratory confirmed that the isolate was resistant to ceftriaxone (MIC = 0.25 mg/L) and had reduced susceptibility to azithromycin (MIC = 1.0 mg/L). The individual was successfully treated with intramuscular (IM) ceftriaxone 1 g plus oral azithromycin 2 g and reported no other recent sexual partners. No further cases were identified in England.

In February 2018, the first *N. gonorrhoeae* strain that was resistant to ceftriaxone (MIC = 0.5 mg/L) and also had HL-AziR was reported in England in a heterosexual male with urethral and pharyngeal infections acquired in Thailand [19]. PHE issued an international alert via the Early Warning and Response System of the European Union (EWRS). The urethral infection was successfully treated with ceftriaxone (1 g), but the pharyngeal infection persisted and was eventually cleared after 3 days treatment with intravenous (IV) ertapenem 1 g. After the patient's regular female partner based in England tested negative for gonorrhoea, it was concluded that the resistant strain was unlikely to have spread further in England. Primary diagnostic laboratories were reminded to refer *N. gonorrhoeae* isolates with suspected ceftriaxone resistance (MIC > 0.125 mg/L) to the national reference laboratory for confirmation. Shortly after, in April 2018, Australia notified the WHO of two isolates of the same strain of *N. gonorrhoeae*, prompting the ECDC to release a rapid risk assessment [20]. Since then, the ECDC has also updated their 2012 Response Plan for managing AMR gonorrhoea in Europe [21].

Later in 2018, two women in England were diagnosed with *N. gonorrhoeae* infections caused by strains with ceftriaxone resistance (MIC = 1.0 mg/L) and reduced azithromycin susceptibility (MIC = 0.5 mg/L) [22]. The use of WGS confirmed that these infections were caused by the FC428 clone. The diligent investigation by local sexual health clinicians identified an epidemiological link to the same sexual network in Ibiza, Spain for both women. The ECDC was alerted via EWRS, and PHE disseminated briefing notes to laboratories and to the BASHH clinical network. One woman, whose partners were untraceable, was successfully treated with IM ceftriaxone 500 mg and oral azithromycin 1 g. The second woman was treated successfully with 3 days of IV ertapenem 1 g (ertapenem MIC = 0.032 mg/L) after firstly IM ceftriaxone 1 g and then IM gentamicin 240 mg (gentamicin MIC = 4.0 mg/L) plus oral azithromycin 2 g had failed to clear her infection. A partner of the second woman tested positive for pharyngeal gonorrhoea and was treated with IV ertapenem 1 g for 3 days empirically, returning for a negative test-of-cure 2 weeks later.

In 2019, three further cases of ceftriaxone-resistant (MICs: 0.5–1 mg/L) gonorrhoea were detected in England [15]. All were associated with travel from China and no mutual partners were identified. As there was no evidence of acquisition or transmission within England and all cases were successfully treated with 1 g ceftriaxone, a risk assessment concluded that no further public health actions were required. No cases of ceftriaxone-resistant gonorrhoea were identified in England in 2020, a year when international travel was largely restricted in response to the COVID-19 pandemic.

Close collaboration between sexual health services, local laboratories and the UKHSA was essential to identifying that all cases of ceftriaxone-resistant isolates reported in the UK since 2013 had been linked to international travel, mostly from the Asia-Pacific region. These data informed the updated 2019 UK guideline for managing infection with *N. gonorrhoeae*, which recommended multi-site testing for those who may have acquired gonorrhoea in the Asia-Pacific region and prioritised test-of-cure for returning travellers with gonorrhoea [11].

## Development of surveillance systems

In 2011, the HPA (now UKHSA) launched an encrypted online tool with restricted access to report suspected cases of gonorrhoea treatment failure. All sexual health clinicians in England were requested to report all gonorrhoea cases failing first-line treatment, by accessing the online reporting system. Distinguishing between reinfection, false-positive molecular tests detecting residual DNA at test-of-cure and genuine treatment failure remains a challenge for clinicians; as a result, UKHSA follows up and provides assistance in investigating and following up all possible cases. UKHSA also continues to raise awareness of this reporting system through clinical networks and feeds back findings from this reporting in the annual GRASP reports.

GRASP collects isolates during a 3-month period every year from a representative sample of clinics, which covers around 2% of all gonorrhoea diagnoses in England, and reports are published a year later. Since 2015, GRASP data have been supplemented by real-time laboratory data to enable the rapid detection of emerging AMR. The Second-Generation Surveillance System (SGSS) was launched in 2014 and captures routine data on infectious diseases and AMR from all primary diagnostic laboratories across England on a weekly basis; reporting of sexually transmitted infections is voluntary. The data collected through SGSS are more timely and have much broader coverage than those collected through GRASP. However, SGSS does not include any behavioural or clinical information and reports antibiotic resistance categories (susceptible or resistant) but no MIC data. National guidelines recommend that all patients with gonococcal infection should have a sample taken for culture before commencement of treatment [11], and that all *N. gonorrhoeae* isolates should be tested for ceftriaxone susceptibility and referred to the national reference laboratory if suspected to be resistant. However, only seven of 42 isolates reported in SGSS with suspected ceftriaxone resistance were referred to the reference laboratory in 2020. Of some reassurance, the majority of referred isolates were not confirmed as resistant by the reference laboratory (0/7 in 2020) [10]. UKHSA carried out 6 months of enhanced surveillance of *N. gonorrhoeae* isolates that were reported in SGSS as either not tested for susceptibility or with suspected ceftriaxone resistance. While most isolates with suspected resistance had been incorrectly reported, mainly due to transcription errors, the surveillance exercise demonstrated that awareness of the national guidelines required strengthening, and it is possible that cases of ceftriaxone-resistant gonorrhoea have been missed. As of 2021, UKHSA continues to review SGSS data from primary diagnostic laboratories on a weekly basis and contacts laboratories that have reported a ceftriaxone-resistant *N. gonorrhoeae* isolate to verify that it has been sent to the national reference laboratory for confirmation. Any isolates that are confirmed as ceftriaxone-resistant initiate a rapid risk assessment.

In 2019, there were reports of a cluster of disseminated gonococcal infection (DGI) in the United States [23]. In 2020, UKHSA therefore initiated enhanced surveillance for DGI to determine whether specific public health actions were needed. Previously, DGI was estimated to occur in 0.5 to 3% of individuals with untreated gonorrhoea, but there are no recent data for its incidence in England. Preliminary surveillance data demonstrated that at least 11 cases of confirmed or probable DGI were identified in England from 2016 to 2020, which is considerably less than 0.5–3% [24]. However, retrospective reporting is likely to lead to an under-estimation of the true number of cases. Most cases were reported among GBMSM, the median age of cases was 27 years, and nearly all cases were hospitalised, for a median duration of 8 days.

## Conclusion

The emergence of highly resistant *N. gonorrhoeae* strains in recent years is of worldwide concern. UKHSA are currently reviewing and updating the GRASP 2013 Action Plan to reflect the changes that have been made to strengthen real-time surveillance of gonococcal AMR and to include the lessons that have been learnt from the management of AMR *N. gonorrhoeae* incidents. Continued engagement of sexual health clinicians and diagnostic laboratories with the UKHSA is essential for the timely detection of *N. gonorrhoeae* strains with resistance to ceftriaxone and to rapidly contain transmission of these strains within England.
